# Defecation of a “colon cast” as a rare presentation of acute graft-versus-host disease

**DOI:** 10.4103/0256-4947.51783

**Published:** 2009

**Authors:** Hamad Al Ashgar, Musthafa Peedikayil, Naeem Chaudhri, Abdulmonem Al-Ghamdi

**Affiliations:** a From the Department of Medicine, King Faisal Specialist Hospital and Research Centre, Riyadh, Saudi Arabia; b From the King Faisal Cancer Centre, King Faisal Specialist Hospital and Research Centre, Riyadh, Saudi Arabia; c From the Department of Pathology and Laboratory Medicine, King Faisal Specialist Hospital and Research Centre, Riyadh, Saudi Arabia

## Abstract

Diffuse involvement of the gastrointestinal tract by graft versus host disease (GVHD) is a common complication of allogeneic hematopoietic stem cell transplant (HSCT). Gastrointestinal GVHD usually presents 3 or more weeks after HSCT and is characterized by profuse diarrhea, anorexia, nausea, vomiting, abdominal pain and gastrointestinal bleeding. We report a case of a 23-year-old male who had undergone allogeneic HSCT and presented with bloody diarrhea on the 90th day post-HSCT. On the fourth day of admission, the patient passed per rectum a 27-cm long pinkish colored fleshy material recognized as a “colon cast”. Sigmoidoscopy showed a congested and erythematous rectum with the remaining portion of the “colon cast” attached to the proximal part of the sigmoid colon. A biopsy from the rectal wall was suggestive of grade IV GVHD. The patient was treated with methylprednisolone, cyclosporin and mycophenolate mofetil, with a partial response (diarrhea and abdominal pain improved), but then he developed multiple other medical complications and died after 3 months.

Acute graft-versus-host disease (GVHD) occurs after allogeneic hematopoietic stem cell IA transplantation (HSCT) as a consequence of an immune reaction by the donor immune cells against host tissues. About 35% to 50% of HSCT recipients develop acute GVHD.^1^,^2^ Clinically, the diagnosis is suspected when a recipient of HSCT develops any or all of the following signs or symptoms: dermatitis (skin rash), cutaneous blisters, crampy abdominal pain with or without diarrhea, persistent nausea and vomiting, or hepatitis (with elevation of bilirubin and/or liver enzymes).^3^ Typically, these symptoms occur before day 100 after the HSCT, but may occur later.^4^

The spontaneous passage of a full-thickness colon cast has been described eight times previously,^5^–^9^ including one case of a mucosal cast. In five cases the etiology was occlusion of the mesenteric artery. In the remaining three cases, one patient had pancreatitis, the other had undergone resection of a lower rectal carcinoma with a sigmoid pouch–anal anastomosis and ileostomy, and the last had undergone Hartmann's procedure sacrificing the inferior mesenteric artery for a perforated diver--ticulum and pelvic abscess.^5^–^9^ To our knowledge, there are no reports of spontaneous passage of a colon cast in the context of acute GVHD.

## CASE

**Figure 1:**
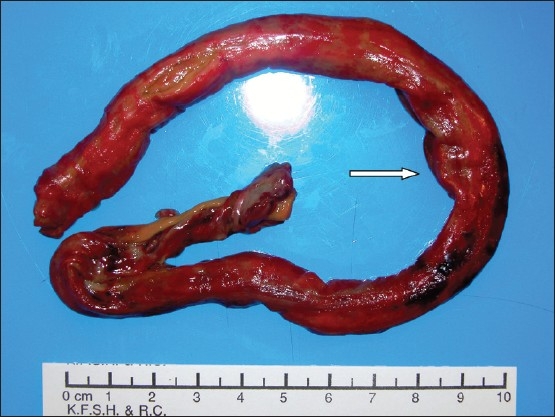
Gross appearance of the colon cast passed per rectum.

A 23-year-old male underwent allogeneic HSCT for aplastic anemia 90 days prior to presentation. The patient presented to the emergency department with a history of severe lower abdominal pain, bloody diarrhea and fever of 3-days duration. He was discharged from the hospital a few days prior to this presentation after recovering from multiple medical problems following HSCT including E *coli* and *Streptococcus viridans septicemia,* febrile neutropenia, cytomegalovirus (CMV) antigenemia, GVHD involving the skin, and aspergillosis of the lung. On admission the patient was febrile and appeared pale. The skin was hyperpigmented from GVHD. The oral cavity showed features of mucositis. The lower abdomen was tender on palpation. The cardiovascular, respiratory and nervous system examination was unremarkable. Broad-spectrum antibiotics and other supportive measures were begun and the patient was moved to the intensive care unit. On the fourth day of admission the patient passed a 27-cm long, tubular, pinkish-colored fleshy material rectally (Figure 1). This structure was recognized as a “colon cast” and was sent to pathology for histology. The stool examination for culture and sensitivity of *Clostridium difficle* was negative. A CT scan of the abdomen showed non-specific thickening of the large and small bowel including rectum as well as inflammation of the surrounding of the mesenteric fat. A sigmoidoscope was unable to pass more than 25 cm from the anal verge. The sigmoid and rectal mucosa was congested and erythematous. At 25 cm from the anal verge, the residual part of the “colon cast” was seen attached to the colonic wall.

**Figure 2:**
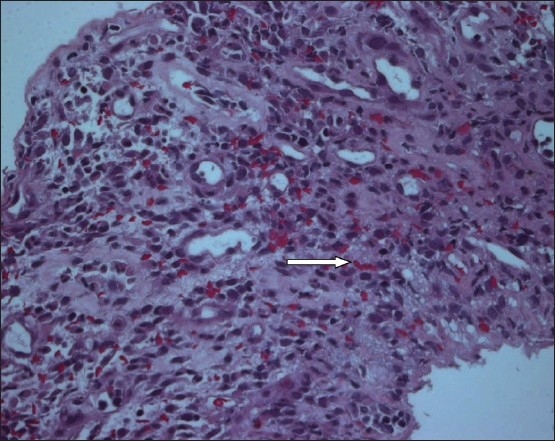
Histology of the sigmoid colon showing granulation tissue and base ulcers suggesting severe graft versus host disease.

**Figure 3:**
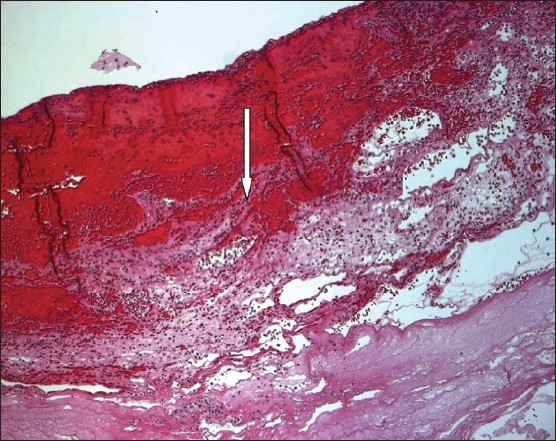
Histology of the “colon cast” showing necrotic fibrino-purulent exudate with bacterial overgrowth.

Laboratory tests on admission were as follows: WBC 7.46×10^9^/L (normal, 3.9-11×10^9^/L), hemoglobin 104 g/L (normal, 110-160 g/L), platelet count 10×10^9^/L (normal, 155-435 10^9^/L), urea 5.3 mmol/L (normal, 2.5-7.5 mmol/L), creatinine 88 micromols/L (normal, 46-96 micromols/L), potassium 3.1 mmol/L (normal, 3.5-5 mmol/L), sodium 138 mmol/L (normal, 135-147 mmol/L), carbon dioxide 19 mmol/L (normal, 22-31 mmol/L), albumin 26 g/L (normal, 32-48 g/L), bilirubin 21 μmol/L (normal, 0-21 μmol/L), lactic acid 0.8 mmol/L (normal, 5-2 mmol/L), ALT 30 U/L (normal, 10-45 U/L), and alkaline phosphatase 223 U/L (normal, 30-125U/L). The histology of the sigmoid colon showed granulation tissue and base ulcers suggestive of grade IV acute GVHD (Figure 2). The histology of the colon cast showed necrotic fibrino-purulent exudate with bacterial overgrowth (Figure 3). There was no evidence of CMV infection in the histology of the sigmoid colon and CMV culture was negative.

The patient was treated for acute GVHD with methylprednisolone, cyclosporine and tacrolimus. He was later treated with mycophenolate mofetil and anti-thymocyte globulin as per our hospital protocol. Appropriate antibiotics, hydration and other supportive measures were also given. The patient showed some response to treatment with a reduction in the amount of diarrhea. Unfortunately, the patient developed multiple complications afterwards, including gram-negative sepsis, CMV infection, and aspergillosis of the lung, progressive pancytopenia and graft failure. The patient died in the hospital due to multi-organ failure 3 months after admission.

## DISCUSSION

About 35% to 50% of HSCT recipients will develop acute GVHD.^10^ Acute GVHD is a clinical diagnosis, but other conditions can mimic or coexist with GVHD, such as drug toxicity and infections. Therefore, a biopsy is recommended to confirm clinical suspicion whenever possible.^11^

The pathophysiology of acute GVHD is a complex process. Patients who undergo bone marrow transplantation receive high-dose chemotherapy before the procedure. High-dose chemoradiotherapy damages the host tissues causing release of inflammatory cytokines such as tumor necrosis factor (TNF)-alpha and interleukin 1, which lead to activation of host antigen-presenting cells. The donor T-cells recognize alloantigens on host antigen-presenting cells and activate the donor T-cells. The activated T-cells then proliferate and differentiate into effector cells and secrete cytokines such as interferon (IFN)-gamma. TNF-alpha and IFN-gamma cause further injury to the gastrointestinal epithelium leading to the tissue damage characteristic of acute GVHD.^12^

The gastrointestinal system is the third most common organ involved in acute GVHD. Gut GVHD may involve any part of the gastrointestinal tract and is frequently the most severely involved organ. It is often difficult to treat gut GVHD. Diarrhea and abdominal cramping are generally the hallmarks of gut involvement. Clinical manifestations of gut GVHD include diarrhea, nausea, vomiting and crampy abdominal pain. There may be a large volume of diarrhea, which is often bloody. Abdominal distention and paralytic ileus may develop.^13^ Many of the gastrointestinal symptoms are nonspecific and hence endoscopic biopsy confirmation is often needed.

Upper and lower gastrointestinal endoscopy is a useful modality to diagnose acute GVHD and to differentiate it from other causes of diarrhea. Endoscopic findings of gut GVHD may vary from normal to extensive edema, mucosal sloughing or diffuse bleeding from the gut mucosa. The endoscopy may show prominent lesions in the cecum, ileum, and ascending colon. The mucosa involvement can be seen in the stomach, duodenum, and rectum as well.^13^

Endoscopy is a useful tool to grade the severity of acute GVHD, helping the physician to choose appropriate treatment and to predict prognosis. Endoscopic grading is based on the appearance of colonic mucosa during colonoscopy or sigmoidoscopy. Endoscopically, acute GVHD can be classified as stage 1 to 4. Stage 1 is normal appearing mucosa, stage 2 will show a loss of vascular marking of normal colonic vascular pattern or there will be focal mild erythema. Stage 3 acute GVHD will have colonic mucosal edema, erythema, erosions, and/or bleeding. Stage 4 is characterized by colonic ulceration and bleeding.^14^ The biopsy is also highly useful for assessing the severity of acute GVHD. Histological grading of GVHD is also divided into 4 classes. Grade 1 GVHD is characterized by increased crypt apoptosis; grade 2 with apoptosis and crypt abscess; grade 3 with individual crypt necrosis; and grade 4 by total denudation of areas of the mucosa.^15^ According to the above classification of assessment of severity of gut GVHD, this patient had stage 4 and grade 4 acute GVHD.

To our knowledge, passage of the colonic cast as a manifestation of acute GVHD has never been reported. Previously, one case was reported in the literature, with passage of esophageal mucosal cast as a complication of chronic GVHD following bone marrow transplantation.^16^ We propose that the colon mucus cast in this patient was the result of severe GVHD, leading to a total denudation of the mucosa from the deeper layers of the colonic wall.
